# Increasing uptake to a lung cancer screening programme: building with communities through co-design

**DOI:** 10.1186/s12889-022-12998-0

**Published:** 2022-04-23

**Authors:** Lynsey Rachael Brown, Frank Sullivan, Shaun Treweek, Anne Haddow, Rodney Mountain, Colin Selby, Mara van Beusekom

**Affiliations:** 1 School of Medicine, University of St Andrews, North Haugh, St Andrews, Scotland, UK KY16 9TF; 2 Health Services Research Unit, University of Aberdeen, Foresterhill, Aberdeen, Scotland, UK AB25 2ZD; 3 Fife Community Advisory Council (PPI), Fife, UK; 4 Ninewells Hospital, NHS Tayside, Dundee, Scotland, UK DD1 9SY; 5 NHS Fife, Fife, UK; 6 Dutch Centre of Expertise On Health Disparities, Pharos, Netherlands

**Keywords:** Cancer screening, Lung cancer

## Abstract

**Background:**

Lung cancer is the most common cause of cancer death in the UK. Low-dose computed tomography (LDCT) screening has been shown to identify lung cancer at an earlier stage. A risk stratified approach to LDCT referral is recommended. Those at higher risk of developing lung cancer (aged 55 + , smoker, deprived area) are least likely to participate in such a programme and, therefore, it is necessary to understand the barriers they face and to develop pathways for implementation in order to increase uptake.

**Methods:**

A 2-phased co-design process was employed to identify ways to further increase opportunity for uptake of a lung cancer screening programme, using a risk indicator for LDCT referral, amongst people who could benefit most. Participants were members of the public at high risk from developing lung cancer and professionals who may provide or signpost to a future lung cancer screening programme. Phase 1: interviews and focus groups, considering barriers, facilitators and pathways for provision. Phase 2: interactive offline booklet and online surveys with professionals. Qualitative data was analysed thematically, while descriptive statistics were conducted for quantitative data.

**Results:**

In total, ten barriers and eight facilitators to uptake of a lung cancer screening programme using a biomarker blood test for LDCT referral were identified. An additional four barriers and four facilitators to provision of such a programme were identified. These covered wider themes of acceptability, awareness, reminders and endorsement, convenience and accessibility. Various pathway options were evidenced, with choice being a key facilitator for uptake. There was a preference (19/23) for the provision of home test kits but 7 of the 19 would like an option for assistance, e.g. nurse, pharmacist or friend. TV was the preferred means of communicating about the programme and fear was the most dominant barrier perceived by members of the public.

**Conclusion:**

Co-design has provided a fuller understanding of the barriers, facilitators and pathways for the provision of a future lung cancer screening programme, with a focus on the potential of biomarker blood tests for the identification of at-risk individuals. It has also identified possible solutions and future developments to enhance uptake, e.g. Embedding the service in communities, Effective communication, Overcoming barriers with options. Continuing the process to develop these solutions in a collaborative way helps to encourage the personalised approach to delivery that is likely to improve uptake amongst groups that could benefit most.

## Background

Lung cancer is the most common cause of cancer death in the UK and globally [1, 2]. 48,000 people are diagnosed in the UK each year, of these 80% have a poor prognosis, with the 5-year survival rate at just over 15% [1]. Lung cancer is often diagnosed late in the disease trajectory [3], with a significant number being diagnosed as an emergency [4]. There is a need for effective means of earlier diagnosis to enable curative treatment and improve survival [5]. Individuals at higher risk of developing lung cancer include smokers and ex-smokers, those living in areas of deprivation and those aged ≥ 50 [1]. These groups also experience poorer outcomes [1, 6–9], exemplifying the impact of the inverse care law [10]. Screening for lung cancer, targeted to those who are at greater risk, could ameliorate this disproportionate effect.

Previous research on lung cancer screening has shown the benefits of low dose computed tomography (LDCT), enabling diagnosis at an earlier stage and reducing lung cancer mortality in those aged 50 to 74 years who are past and current smokers [11–13]. However, questions around risk vs benefit, available resources and cost-effectiveness [14–18] need to be resolved before LDCT can be used as an effective tool to screen for lung cancer. To ensure that the benefits outweigh potential harms, LDCT screening should be targeted to those who are at particularly high risk of developing lung cancer. To do this, trials have focussed on the use of tools to assess risk before patients undergo LDCT, such as the UKLS First Approach Questionnaire [19], Prostate, Lung, Colorectal and Ovarian (PLCO) questionnaire [20] and Liverpool Lung Project (LLP) questionnaire [21]. To increase uptake, lung health checks piloted in England offer LDCT immediately after presenting the risk score and in convenient locations, with promising results [22].

Uptake to screening trials, which might influence service provision, is often poor and generally ranges between 25 and 50% [22, 23] but can be as low as 3–4% for lung cancer screening in the United States [15]. Those at greater risk from lung cancer are often underrepresented in screening trials [24], while smokers and those from more deprived areas have also been found to be more likely to decline an invitation to screening [25].

There are also concerns about the ability of questionnaire-based risk prediction tools to determine accurately the level of risk and who should be referred for LDCT [26]. To ameliorate these issues and enable a systematic approach to risk prediction a move towards autoantibody biomarker blood tests could be considered, with Ouderk et al. [16] indicating this as a next step in risk prediction. Blood tests or other biomarkers could help systematically identify those at high risk for lung cancer and therefore, LDCT screening [27–30]. Further research is needed regarding the effectiveness of biomarkers before they are implemented widely to ensure sensitivity and specificity.

To improve uptake and the likely success of a biomarker blood test, as part of a lung cancer screening programme, further preparatory research is needed. Among other things, insight is needed regarding specific barriers and facilitators of uptake and provision, as well as potential pathways for provision and participation. Recently there has been calls for the co-development of interventions designed to improve uptake of lung cancer screening in deprived areas where the disease incidence is highest [31, 32]. Active contributions to problem identification and solving from both those potentially providing and using the test enables the development of a service designed around the needs of those who will benefit most [33], leading to a suitable service.

## Methods

The aim was to identify ways to further increase opportunity for uptake of a lung cancer screening programme, using a biomarker blood test for LDCT referral, amongst people who could benefit most. Targeting those aged 55 and over, who smoke or have smoked in the past and are living in areas of deprivation. A 2-phase co-design process following the double diamond model of co-design [34], moving from discovery and defining the problem, to developing solutions and delivery was used.

Ethical approval was granted by the University of St Andrews, School of Medicine Ethics Committee (Approval code: MD14677).

### Recruitment and participants

#### Group 1: members of the public

Members of the public were recruited through community groups, including: a local football club, a pigeon fancier group, a veterans (ex-military and older) group and a local peer support group, with leads acting as gate keepers, targeting those aged ≥ 55, (ex-)smoker and living in higher areas of deprivation.

#### Group 2: professionals

A targeted approach was used to recruit professionals in services that could provide the test in the future and/or where individuals were working closely with the target group.

### Phase 1

#### Process

A face-to-face focus group was conducted with members of the public (*n* = 7), with a graphic artist present to visually capture the discussion. A further nine individual interviews with the public were carried out by telephone. Nine individual interviews and one joint interview were conducted with professionals (*n* = 11) using Microsoft Teams. Interviews and focus groups lasted approximately one hour and were facilitated by 1–2 members of the research team.

### Materials

#### Topic guide 1: members of the public

**Fig. 1:**
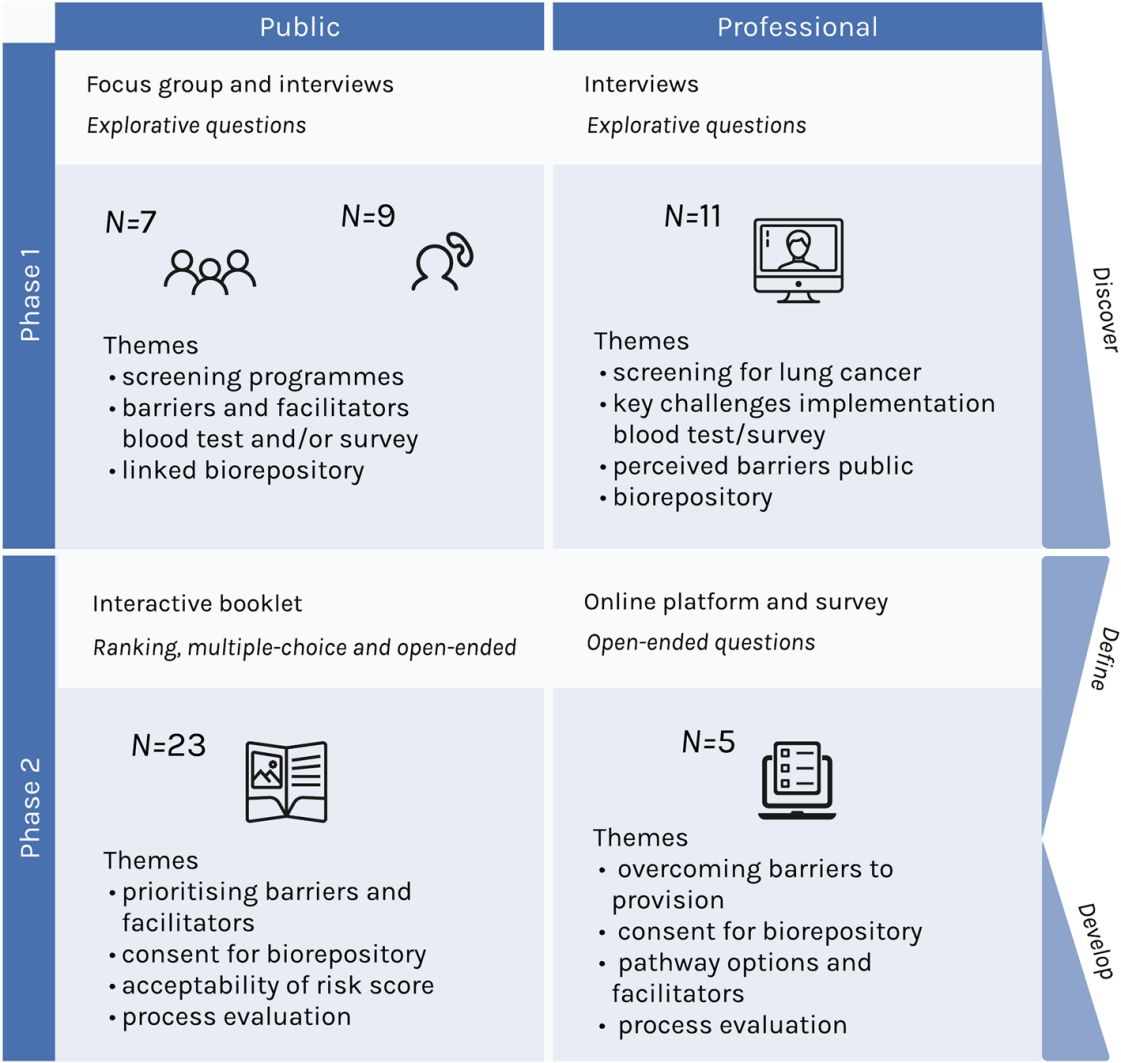
Discussion content Phases 1 and 2

This topic guide focussed on previous experiences of and views on screening programmes, barriers and facilitators to participation in a screening programme for lung cancer using a blood test and/or survey, as well as views and possible concerns of consenting to a biorepository (Fig. 1).

#### Topic guide 2: professionals

This topic guide focussed on views of screening for lung cancer, key challenges in implementing a screening programme in practice-incorporating a blood test and/or survey, perceptions of barriers the public may face in participating, as well as views and possible concerns of consenting patients to a biorepository in conjunction with the screening (Fig. 1).

### Analysis

An initial mapping session was run with the research team to discuss the data and consider the key pathways, barriers and facilitators arising from the data. Audio-recordings were transcribed verbatim and analysed using thematic analysis [35]. Double coding was conducted on 20% of the data to assess interrater reliability. Cohen’s Kappa co-efficient score of 0.69, indicated moderate to excellent reliability of the codebook (NVivo 12). Themes were further mapped to previously proposed factors affecting uptake: Acceptability, Convenience & Accessibility, Awareness and Reminders [36].

### Phase 2

#### Process

An interactive offline exercise and online survey were implemented with members of the public and professionals respectively, drawing on the results from Phase 1.

#### Materials

##### Offline interactive exercise: members of public

**Fig. 2:**
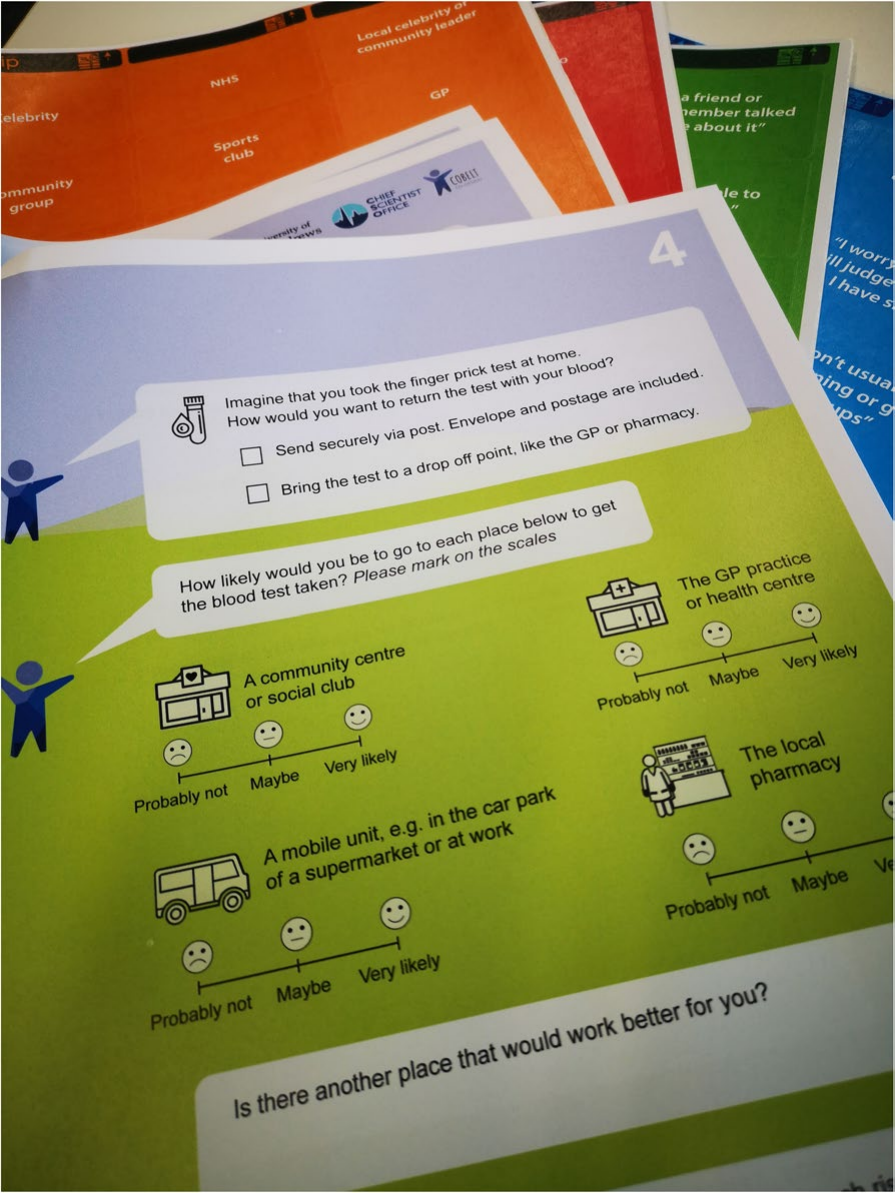
Example materials phase 2 (interactive booklet)

Drawing on the tenets of cultural probes [37], packs were created consisting of a booklet with 19 open/closed topical questions, six evaluation questions and eight demographic questions. Also included were a sticker pack and 0.6 ml microtube to help make the volume of blood needed for the blood test more tangible (Fig. 2). The booklet focussed on prioritising barriers, facilitators, endorsements and advertisement means, drawing on the findings from Phase 1. In addition, it prompted further exploration of potential solutions. It was designed as an easy to read, offline format to minimise barriers to participation. The sticker packs consisted of four categories of responses, 1: Barriers to taking the test, 2: Facilitators to taking the test, 3: Ways to hear about the test and 4: People to hear about the test from, generated from Phase 1 to help relay answers (Fig. 1).

##### Online qualtrics survey/padlet: professionals

Professionals were provided with two options for engagement at this phase to increase the likelihood of participation. A link to an interactive online platform where participants can interact with each other (Padlet) and an individual survey (Qualtrics) was provided, both with the same information and questions. Questions on prioritisation and means to overcome barriers and facilitators to uptake/provision identified in Phase 1 were used, as well as questions building on potential solutions to issues raised (Fig. 1).

Both public and professional materials had an embedded evaluation section to ascertain the acceptability of the methods used.

##### Analysis

As materials collected both quantitative and qualitative data a combined approach to analysis was conducted. All data was extracted from the surveys, collated and input to Excel. Qualitative findings were added to the thematic analysis of Phase 1. Descriptive statistics were conducted for the quantitative data. To achieve an overall rank score for barriers, facilitators, and endorsements/reminders individual scores were multiplied by their rank (1^st^x3, 2^nd^x2, 3^rd^x1) and then summed.

## Results

### Participants

#### Phase 1

##### Group 1

*N* = *16.* Participants were primarily male (*N* = 15), from areas classed as the lower three quintiles of the Scottish Index of Multiple Deprivation (SIMD: *N* = 10), ex/smokers (*N* = 14) and aged 51–86 (Table 1).

**Table 1:** Demographics (*One participant 28y/o as all members of a local group took part)

**Phase 1**	
**Variable**	***N* = 16**
**Gender n (%)**	
Male	15 (94%)
Female	1 (6%)
**Age**	
Mean	66
Range	51–86
**History of lung disease n (%)**	
Yes	3 (20%)
No	13 (80%)
**Lung cancer in family n (%)**	
Yes	0 (0%)
No	15 (94%)
Unsure	1 (6%)
**Smoking status n (%)**	
Smoker	2 (12.5%)
Ex-smoker	12 (75%)
Non-smoker	2 (12.5%)
**Pack years**	
Mean	25
Range	1–55
**SIMD Quintile n (%)**	
Quintile 1–2	5 (31.25%)
Quintile 3	5 (31.25%)
Quintile 4–5	6 (37.5%)
**Phase 2**	
**Variable**	***N* = 23**
**Gender n (%)**	
Male	12 (52%)
Female	11 (48%)
**Age**	
Mean	58
Range	28*-75
**History of lung disease n (%)**	
Yes	7 (30.5%)
No	15 (65.2%)
Missing	1 (4.3%)
**Lung cancer in family n (%)**	
Yes	3 (13%)
No	19 (82.7%)
Missing	1 (4.3%)
**Smoking status n (%)**	
Smoker	6 (26%)
Ex-smoker	10 (44%)
Non-smoker	7 (30%)
**Pack years**	
Mean	28
Range	4–63
**SIMD Quintile n (%)**	
Quintile 1–2	10 (43.5%)
Quintile 3	3 (13%)
Quintile 4–5	5 (21.7%)
Missing	5 (21.7%)

##### Group 2

*N* = 11 professionals participated, including two GPs, two practice nurses, two community pharmacists, two respiratory consultants, one consultant radiologist and two community service leads.

### Phase 2

#### Group 1

A total of 23 members of the public participated – 6 in a group setting facilitated by the group’s support worker and 17 individually. 12 participants were male, most were from areas classed as the lower three quintiles of the SIMD (*N* = 13), ex/smokers (*N* = 16) and aged 28–75 (Table 1).

#### Group 2

A total of 5 professionals participated in Phase 2, 2 GPs, 1 Consultant radiologist, 1 Pharmacist and 1 community links practitioner. 4 of the 5 participants who took part in Phase 2 also took part in Phase 1.

### Barriers & facilitators to uptake of test

Ten barriers and eight facilitators to uptake were identified through the thematic analysis procedure of Phase 1 and expanded on in Phase 2. These were further categorised into Acceptability, Convenience & Accessibility, Awareness and Reminders (Table 2).

**Table 2:** Barriers and facilitators to uptake and provision, with evidence

**Barriers to uptake**			**Facilitators to uptake**		
**Acceptability**					
B1	Fear of result and impact	*“So, some people might have a fear of the unknown… they just don’t want to know and the fear of knowing and where do we go from here and what’s happens to me, ken what happens to them as an individual then.” (Public, male, 60y/o)*	F1	Perceived benefits	I1: “Preventions better than cure, ae?” I2: “Aye” I3: “Aye prevention that’s the main one.” (Community group discussion) “Me personally, my personal opinion on it would be, I would want to know and I’d want to get the treatment as quick as I could get treatment, that’s my personal opinion.” (public, male, 60y/o)
B2	Guilt & Stigma	“I think there is always the battle to try to engage smokers without guilt or stigma, and we know that there's still a huge amount of that present, and I think if you start saying smokers come and get screened for cancer, that's off putting to a large swathes of the very population we want to capture, and we know there's a stigma attached to it.” (Respiratory consultant)			
B3	Attitude	*“…the apathy of the public or my apathy, I better not just say mine because I can mention my wife and she turned 60 two years and I had to get her told to do her bowel screening, she wasn’t doing it. And it was just purely because she didn’t think it mattered. And how you get round that apathy I do not know.“ (Public, male, 65y/o)*			
B4	Mental health & anxiety	“So, I work with a few people who have got agoraphobia and are scared of leaving the house, so if they absolutely need to go to the 5GP then they will but the chances of them going for a blood test, they’re probably not going to, they’re probably going to say I don’t need to go to that. In their view it’s probably not going to be life or death although actually it could be. Unless they’ve got something really quite severe at that moment in time, the chances of them going are pretty slim, mainly due to their anxiety.” (Community links worker)			
B5	Hesitation around Covid 19	“Yes, I definitely think I would, especially for the COVID thing, you’re not wanting anyone in the practice.” (Public, male, 72y/o)	F2	Trust in HCP	“I think that going to the GP would be the easiest for me. They know what they’re doing and they do it very efficiently, so I’m quite happy with that idea.” (public male, 86y/o) “…someone to, suppose it was a district nurse or a pharmacist, suppose you went along to the pharmacist and the pharmacist showed you how to do it. I think that would be very good for the first time.” (public, male, 65y/o)
B6	Lack of engagement with healthcare	*“… I don’t like to bother my doctors and that, ken, you feel as though you’re a nuisance unless you’re feeling really unwell. So, to do it by myself for myself, I think that’s a great idea, as long as you’re able to do that of course, which I am obviously.” (Public, male, 72y/o)*			
**Convenience/accessibility**					
B7	Lifestyle (keeping appointments)	“So, they might say to yes I’ll go and do that but you don’t know what somebody’s lifestyle is when you’re sending these things out, so if they’ve got a chaotic lifestyle or they’ve got a busy lifestyle they might not turn up at a doctors surgery, if they make an appointment that way or for a nurse or even a pharmacist.” (Public, male, 60y/o)	F3	Immediacy	“but a local pharmacy is a good options, it’s the sort of thing where you can see someone say oh yeah, I’ll just have a go of that, if it can be done there and then. It’s almost the immediacy of it.” (GP)
B8	Travel (mobility & rurality)	“So, yeah and I would say because of the rural communities as well, people might find it hard to travel because well just look at Keith itself, Keith’s just a small town but when you look at Keith on the map it’s quite large but it’s rural, absolutely rural.” (Public, male, 60y/o) “Probably another age group, or group you would need to consider would be elderly people, especially if there was fraility. Ordinarily the GP would do a home visit, as opposed to them coming in to the practice.” (Community Links Worker)	F4	Part of community	R: So this is just talking about the test of course but for the actual test do you think it would also help to have maybe the lung check in places like this? In social clubs or… I2: Aye you could do that, cause you might get a lot more folk coming to it and if there’s someone here to do the test, it’s beneficial to the company as well. Cause you could get a test through the door and just think “aw” but if there’s someone here to do it and there’s other people doing it as well, then you’re gonna do it. Same with the blood test, you can do the blood test at the same time. (Community group discussion)
B9	Literacy	“The thing is, it’s maybe sad to say but there’s all sorts of people in the country that have different levels of education and some people just don’t fully understand what some of the things mean, that’s why it’s got to be in simple terms and well if there’s a helpline to phone so be it.” (Public, male, 65y/o)	F5	Convenience and options	“A visual trigger, like I have to do this but at the same time an option like the if you’re not comfortable doing this at home, then maybe you can get it done somewhere else. And then the option that maybe returning it by post or if you’re more comfortable handing it in to a specific drop off point. I don’t know if it would be possible to do it that way” (Community group discussion)
B10	Number of interactions	*“…if you want to tie it into normal or routine phlebotomy, (…) it's not actually done in practice. (…) They’d need to come and pick [information] up at our practice and then they need to take it to a completely different Health Centre to get the blood sample actually taken, which could create an issue.” (GP)*			
**Awareness**					
			F6	Embed conversations in community	“I think, the more you can make lots and lots of different organisations, so other people who may interact with that person, so the more you can make them aware of it, then the more they are likely to encourage them. So, even talking to the likes of housing associations, food banks, citizen advice bureau, money advice places because they are places supporting those individuals day to day and I think those are the organisations those individuals have a bit more trust in. Obviously they trust the doctor and they listen to the doctor but if they are having day to day conversations with somebody at the foodbank or someone like their housing officer or someone else from the housing, and they just spark up a conversation about it, I think that’s actually just as worthwhile, as a poster up in the health centre for example.” (Community links worker)
			F7	National/targeted campaign	“Yeah, the tele is good, I’m not really in to social media, I don’t want to get involved with that but definitely the radio and tele is quite a big thing, aye and you could do it on local tele as well, you don’t have to do it nationally. Anything that can bring it up, I don’t know about posters or that I dunno whether people look at things, not so sure about that one. But definitely the radio and maybe even in the papers, the local papers.” (public, male 72y/o) “…lung cancer is a big issue around here, since it’s an area of high deprivation and certainly patients are very aware of the possibility of lung cancer because lots of them smoke and things like the Alex Ferguson campaign advert about if you ever cough for more than 4 weeks, come and see your GP and get a chest x-ray, and that picked up a lot of interest in our patients.” (Practice nurse)
**Reminders and Endorsements**					
			F8	Trusted sources	“I guess you’d be writing to them or maybe, so sometimes we’ve been involved in studies and the practice sends out the letter. So you give us the letter and we send out the letter on your behalf so it looks like it’s coming from us, a personal recommendation from us and I think that helps that they know us.” (GP)
	**Barriers to provision**			**Facilitators to provision**	
**Acceptability**					
PB1	Staff capacity (Time & workload)	“There's a big concern about the big workload on physicians so, and partly because of other diseases that may come to the fore because of CTs that are done.” (Respiratory Consultant)	PF1	Buy-in to blood test & screening	*“so, I think it was just a whole bunch of people coming together that were quite enthusiastic and also because everybody believed what we were doing. So, that helps that you buy in to the product, as opposed to cynically doing it because you have another project to do.”* (Consultant radiologist)
PB2	Complications around Covid 19	“because practices have, we are having to clean our rooms before and after patients and we can’t have lots of people in the waiting room. So, we can’t have lots of clinicians with patients waiting at the same time. So, we’ve only got room for 2 patients to wait.” (GP)	PF2	Incentives	“(in) pharmacies, a lot of things are basically, they're driven through funding so… I will kind of be told try to get some more flu jabs, as we will make more money by doing more flu jabs in a day. That kind of thing.” (Community pharmacist) “You know sometimes there’s a payment per patient or something like that.” (GP)
PB3	Attitudes to blood test & uptake	*“It’s really difficult engaging them to come in for that (cervical smear), no matter what we do… Yep, it’s terrible in this area for people not taking up screening to be honest with you. It’s offered to them on a plate.”* (Practice nurse)			
PB4	Hesitations about logistics and follow up	“And the barriers really going forwards because obviously going forwards is slightly different than what it was. It is how the capacity and obviously there are lots of different bits to this but I can speak to the radiology… and it’s just continuously depressing data but the Scottish data, there is 1 in 3 radiology jobs are unfilled.” (Consultant Radiologist)			
**Convenience/Accessibility**					
			PF3	Streamline process and ensure capacity	“We use a computer system in greater glasgow called ordercoms, so that we go on to, so the computer system we use you can tick whatever blood test you want. And if it was on there, you could tick that, so that would be milli seconds.” (GP)
			PF4	Training	*“I think the only thing would be training around lung cancer, because especially in community pharmacy we work a lot more with business numbers and targets and things and less clinical…”* (Community pharmacist)

#### Acceptability

Participants viewed the blood test and lung cancer screening more generally as a positive development. The majority of participants emphasised “prevention is better than cure” and understanding of the benefits of an early diagnosis to improve treatment outcomes. Key facilitators for acceptability of an early-detection programme using a blood test were: Perceived benefits (F1) towards screening and benefits of early-detection and Trust in Health Care Professionals (HCPs) (F2). However, several barriers concerning the acceptability also came to light, *i.e.:*

- Fear of result and impact (B1) Guilt & stigma around smoking (B2).

- Attitude/apathy (B3) Mental health and anxiety (B4).

- Hesitation around Covid-19 (B5) Lack of engagement with healthcare (B6).

- Supporting quotes for the individual themes can be found in Table 2. The following section highlights complex juxtapositions of interacting barriers and facilitators.

#### Perceived benefits vs fear of result

Members of the public often expressed a positive attitude to screening and the blood test specifically,

“I can’t see any reason why I wouldn’t want to take that (blood test).” *(*Male, 65y/o).

This overall positive attitude was further confirmed when ranking the reasons for taking the test, with the view that “Knowing that lung cancer can be treated if it is caught early” coming out on top as encouraging people to engage with the test (Table 3). However, members of the public also expressed fear of a lung cancer diagnosis and consequently, the result of lung cancer screening. In the ranking exercise of reasons to not take the test, “I’d be scared of the test result” was ranked 1^st^.

**Table 3:** Ranking outcomes from Phase 2

Variable	Ranked 1^st^ (score)	Ranked 2^nd^ (score)	Ranked 3^rd^ (score)
Barrier	I’d be scared of the test result	I don’t like to bother the staff at my GP practice	I’d be worried I do the test wrong (home test kit)
Facilitator	Knowing that lung cancer can be treated if it was caught early	I’d need to know the risks and benefits	If it’s possible to do at home If it was offered at a usual check up If someone like a nurse provided the test (joint 3^rd^)
Awareness & endorsement (who)	NHS	GP	Community group
Awareness & what (what)	TV	Letter	Posters

#### Trust in GP vs Lack of engagement with healthcare

Further contrasting elements of influence centred around individual relationships with healthcare. Throughout the interviews and confirmed in the booklets (Table 3), participants indicated trust in their GP practice to both carry out the blood test and provide information regarding it (Table 2). However, participants also indicated they do not like to bother their GP and can feel like a nuisance in doing so, as evidenced in the theme “lack of engagement with health care” (Table 2). This was confirmed in the ranking exercise, with “I don’t like to bother the staff at my GP practice” being ranked as the second most important barrier to uptake (Table 3).

#### Awareness & reminders

Both members of the public and professional stakeholders raised promoting awareness as key to encourage uptake of the blood test. They recommended building upon the precedent of cervical and bowel screening and referred to the benefits of national advertising campaigns (F7), particularly referring to a past campaign using Sir Alex Ferguson that was associated with earlier detection of lung cancer [38]. In the context of engaging with populations in more deprived areas, local, targeted advertisement (e.g. posters in community pharmacies) and encouraging Conversations embedded in the community (F6**)** were strategies highlighted in the interviews by pharmacists, practice nurse and community links practitioner. In line with this, public participants ranked ‘Community groups’ as the 3^rd^ best person/organisation to raise awareness, after the NHS (1^st^) and GP (2^nd^). Suggested communication mediums to raise awareness were ranked as: 1. “TV”, 2. “Letters” and 3. “Posters”.

Professionals, generally working in primary care (GPs, practice nurses and community links practitioners) also emphasised the importance of embedding reminders throughout the patient pathway and utilising Trusted sources (F8). Key points were a follow up call or text message after a non-response to the invitation letter, with preference for a personalised approach to help engage with harder to reach groups, such as through established trust relationships with a links practitioner or GP practice staff (e.g. Health Care Assistant or Practice Nurse).

#### Convenience & accessibility

**Fig. 3:**
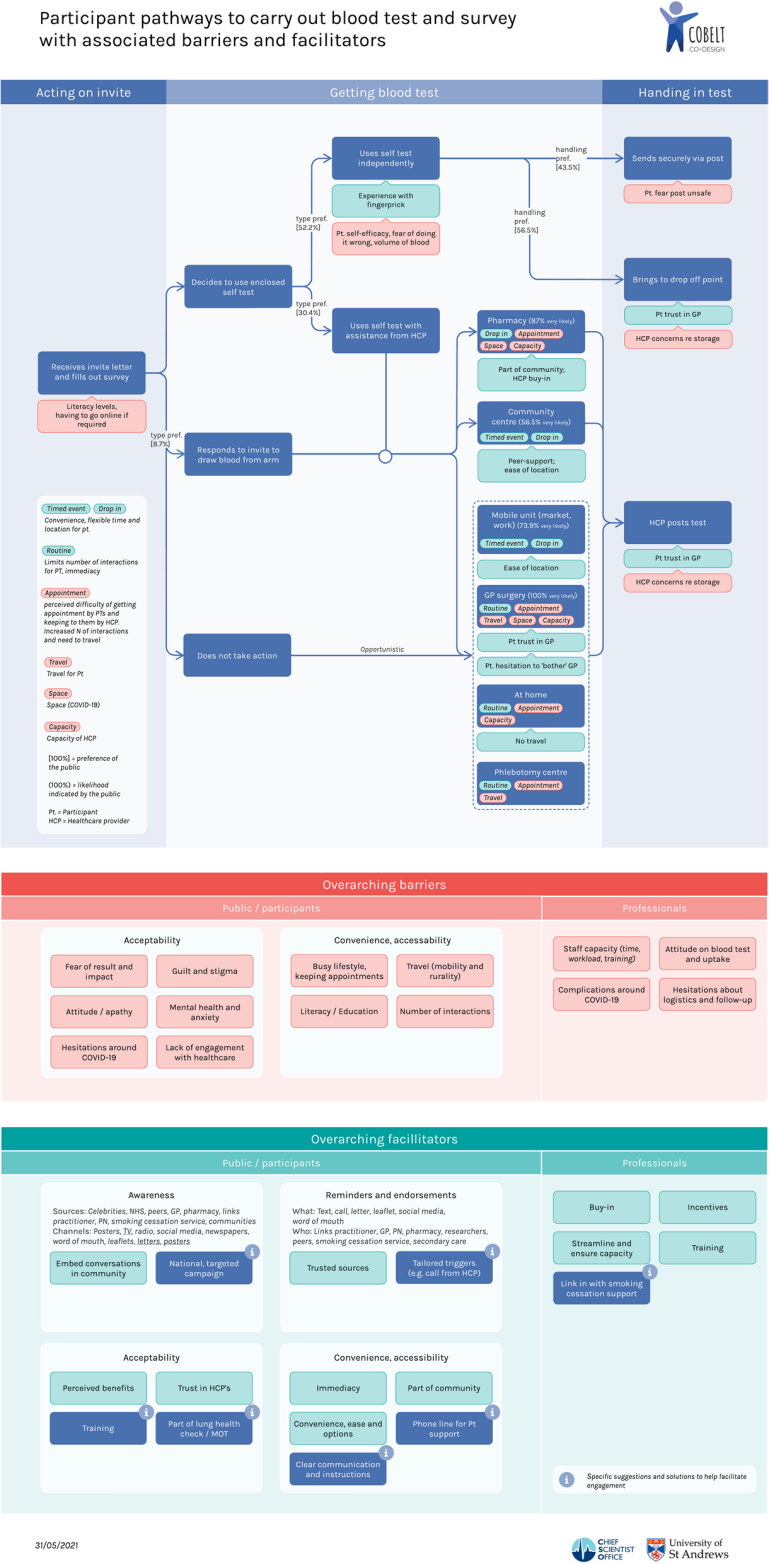
Participant pathways to carry out blood test and survey with associated barriers and facilitators

The majority of barriers and facilitators for the public to engage with the test identified in the conversations represented aspects of convenience and accessibility. Options for delivery of the test centred around a home test kit for the blood test versus a venepuncture test, through a variety of channels and services (Fig. 3-pathway map). Immediacy (F3), Convenience and options (F5), with a specific facilitator being Part of the community (F4), i.e., focus on community centres and groups and again embedding the service in communities. These facilitators were expected to help overcome the barriers associated with convenience and accessibility, including Lifestyle (B7), Travel (mobility and rurality: B8) and Number of interactions (B10). Literacy (B9) was also identified as a barrier emphasising a need for accessible communication.

#### Immediacy

GPs and Community links practitioners emphasised the difficulties some patients may face attending appointments, pointing to existing issues with patients attending scheduled appointments for screening services. Both professional and public stakeholders suggested that "Immediacy" wherein people can be offered the test on the spot, would help to overcome issues with appointments. Pharmacists suggested drop-in clinics to provide this immediacy, indicating offering people appointments may mean they do not return for their appointment, adding to the barrier of number of interactions. Mobile units were also discussed when referring to best practice (Consultants) and means to overcome issues with rurality (Public).

GP practices saw themselves as less suitable for this format but suggested to offer immediacy through offering the test along with routine appointments and recommended a similar approach for phlebotomy services. Integrating delivery of the test with routine appointments was also suggested as a way to overcome the barrier of multiple moments of interaction with the service, which could lead to drop out—e.g. being referred to a phlebotomy service after attending the GP surgery. Smoking cessation services were also suggested as a possible means to promote and/or provide the lung cancer screening programme, embedded within the routine service.

#### Convenience and options

In addition to immediacy, providing different options for the public to engage with the test was identified as a key strategy to promote uptake. Possible options ranged from the GP surgery, home test kit, mobile units (workplace, carparks), community hubs and centres, pharmacies, phlebotomy service and home visits (Fig. 3). Both groups of stakeholders suggested the provision of options could help overcome the range of personal and practical barriers that people may experience. In addition to the location of delivery, three categories of options were discussed: 1. A home test kit versus venepuncture test, 2. Returning the completed blood test, 3. A risk assessment survey versus the blood test.

Nineteen of 21 members of the public preferred a home test kit to getting a venepuncture test, a further 2 did not respond to that question. Pharmacists and primary care staff on the other hand indicated a preference for venepuncture tests as staff are already trained for this and fewer issues or difficulties are attributed to this. Despite the public’s indicated preference for the blood test, some public and professional participants anticipated difficulties with using the home test kit and were worried about getting it wrong. Both of these concerns were ranked highly as influential barriers. Seven of the 19 who preferred the home test kit indicated they would want assistance with the kit. This idea of needing help with the home test kit was also raised in Phase 1. Between Phase 1 and Phase 2 suggestions as to who may provide this help arose, including a friend or relative, an option to stop by the pharmacy for a quick run through, or a helpline to contact for support.

Another potential option was identified for returning the completed blood test. Members of the public evidenced some hesitancy around posting the test back themselves and recommended an option to hand the test in to a trusted location (e.g. pharmacy) as a means to overcome this. In the booklet, 13/23 indicated to prefer this option. Some professional stakeholders were less convinced by this option, with a need for explicit guidance regarding collection and storage.

For the most part discussions regarding the lung cancer screening programme centred on the autoantibody blood test. However, the use of a survey to ascertain risk score was also discussed, highlighting some specific barriers, especially for those at high risk from lung cancer. Members of the public indicated a greater sense of trust in a blood test but generally agreed that taking the survey along with or perhaps before the blood test could be useful. This was corroborated by the booklets in Phase 2, where 13 of 23 agreed that an individual risk assessment would help them decide whether to take the blood test or not.

#### Literacy & communication

Literacy and education levels were also identified as potential barriers impacting accessibility, indicating lower literacy and education levels as a potential influence of engagement and participation in the test. The possibility of a helpline for patients with questions was suggested as a means to overcome this by a member of the public. The impact of low literacy levels was also expected to influence participation in a risk assessment survey. Throughout Phase 1 accessible communication more broadly was also promoted, with members of the public citing the ease of use and understanding of the requirements of the bowel screening programme. Members of the public wanted instructions and information to be clear.

### Barriers & facilitators for provision

In addition to the public-focussed factors that affect test uptake, four main barriers for professionals to provide the test were identified, all of which fall under the acceptability category (Table 2) *i.e.*:

- Staff capacity (PB1)
- Complications due to COVID 19 (PB2)
- Attitude about the blood test and uptake (PB3)
- Hesitations around logistics and follow up (PB4)

Professionals often indicated the already stretched capacity of the service and limited available time to support an additional service. These issues were exacerbated by the COVID 19 pandemic, causing further strain on staff capacity and limited access to facilities for patients. To mitigate these barriers, professionals expressed it was key that the service was Optimally streamlined, ensuring capacity (PF3), referring to an example of team working, whereby everyone knew their role and were invested in the programme. Embedding the programme in current systems was also expected to help with this. Financial incentives (PF2) were suggested as an additional facilitator for pharmacies and GP practices (particularly when running the programmes as a trial).

A third key facilitator to support test delivery was professionals’ Buy-in (PF1) to its benefits, with the effectiveness of screening programmes still being under consideration. Psychological impact was raised often by both professionals and members of the public, in particular the impact of the test result and waiting on referrals and test results. Respiratory consultants emphasised that relevant systems need to be in place for emotional support as well as capacity to facilitate the LDCT follow up in a timely manner, which was a significant concern. Also, the sensitivity and specificity of the test must be considered, with the risk benefit ratio often being cited as something people would want to know. The fourth key facilitator identified by professionals was receiving appropriate Training (PF4) in how to best support patients with this decision-making process, their emotional concerns and queries around lung cancer more generally.

See Fig. 3 for an overview of all pathways, barriers and facilitators identified.

### Perceptions on linked biorepository

Both members of the public and professionals felt that a biorepository associated with the cancer screening programme would be beneficial, citing progress in treatment/research and a desire to help others as reasons. However, respiratory consultants were worried about the feasibility of gathering sufficient tissue. Discussions with professionals also highlighted that those living in more deprived areas may worry about how their data is used:

“My gut feeling is you’d have a lot of people that would say they didn’t want that without having more information about it, they’d be really worried about just out the blue phone calls…” (Community Links Practitioner)

However, most public participants did not feel worried about where their information would be stored.

Three issues were raised around consenting for the biorepository: staff training, time and timing. GP staff expressed a concern about not having the right background knowledge to adequately inform patients and about the time this conversation would add to consultations. A pharmacist indicated that some pharmacies may have more capacity to discuss this. The timing of the conversation was also identified as key – in the interviews, a few members of the public felt that asking for consent together with the blood test was “a lot to swallow in one go.” (Public, male, 72y/o). It was suggested to delay consent for the biorepository until after the result of the blood test or to have the consent already in place through a system similar to SHARE, Scotland’s Health Research Register & Biobank [39], if the patient appeared in clinical practice for the blood test. However, the majority of people (20/23) who completed the booklets indicated they would prefer to consent to the biorepository at the initial invitation.

### Process evaluation

Results from the evaluation embedded in Phase 2 (6 evaluation questions) showed the majority of participants who completed the interactive booklet found it easy to complete (21/23). 22 of 23 found the stickers helpful and 21 of 23 thought the booklet looked good. Most importantly 21 of 23 felt they could contribute, while the remaining 2 felt they could at least contribute a little. Three participants indicated the booklet was too long.

Those who took part in the Padlet or Qualtrics survey were also positive about these formats, indicating both formats were easy to use and provided the opportunity to contribute. One participant noted they had not used Padlet before but had no issues.

## Discussion

The aim of this study was to identify ways to further increase uptake of a lung cancer screening programme using a biomarker blood test for LDCT referral amongst people who could benefit most. A 2-phase co-design process was employed, recruiting both those who may use the programme in the future and those who may provide it. A concerted effort was made to engage those at high risk from developing lung cancer, as these individuals are often underrepresented in lung cancer screening trials and research more generally. Having successfully recruited from this group, this study provides key insights to help design future lung cancer screening programmes, both using biomarker blood tests and more generally, around their needs.

The findings regarding barriers and facilitators to uptake and provision builds and supports those previously identified. Ali et al. [25] also found that low perceived personal risk, practical concerns and emotional concerns were barriers to engage, experienced specifically by those at high-risk of lung cancer. Other reasons for opting out have included misunderstanding, communication, knowledge avoidance, fear, perceived low value and possible worry about false positives [40, 41]. Fatalism, worry, and perceived lack of treatment efficacy were also identified as potential barriers by Quailfe et al. [42] and Smits et al. [43]. Smokers as opposed to non-smokers have been found to be less likely to believe early detection could result in better survival and were less likely to consider CT scans [44]. Moreover, we found Guilt and stigma in smoking behaviour acted as a potential barrier to participation, similar to results of previous studies [41, 45].

As with this study, previous research has also found individuals have an interest in and often intend to attend lung cancer screening. For example, Quailfe et al. found 90% of smokers/ex-smokers intended to attend lung cancer screening [45]. Yet, the uptake remains low. Such discrepancies indicate a potential intention behaviour gap. Some of the means identified to enable attendance and improve uptake could ameliorate this issue, for example “Immediacy”. Providing the test in routine care, enables that immediacy and in turn removes the time available to hesitate between intention and behaviour. Other work has highlighted the importance of immediacy. In the Liverpool Healthy Lung Programme patients were referred to LDCT at a later date, with drop-out being 15% for uptake of LDCT scan [46]. Elsewhere, where the scan was provided immediately after a risk score was obtained, drop-out was less than 5%, including exclusions [22].

### Key priorities for provision to improve uptake

Three key priorities for improving uptake in a future lung cancer screening programme, using a risk indicator for LDCT referral are recommended.

1. Embedding the service in communities
2. Effective communication
3. Overcoming barriers with options.

#### Embedding the service in communities

Embedding the service in communities was offered as a key solution to increase uptake in more deprived areas in particular. An understanding of the community and the impact word of mouth can have in these communities could lead to improved uptake. For example, providing training to services who may interact with the target population regularly could lead to a constant drip of information leading individuals to ultimately participate in the programme. Such embedding would also enable and support further partnership working. There is also potential to work directly with smoking cessation services in a reciprocal manner. Previous research embedding smoking cessation in a lung cancer screening programme has yielded mixed results, with some studies achieving increased quit rates after a positive scan result [47] and others finding no change or poor rates of smoking cessation in both the intervention and control groups [48, 49]. Therefore, further research is needed to understand and develop smoking cessation as embedded within a lung cancer screening programme, as recommended by Minnix et al. [50].

#### Effective communication

In support of embedding the service in the community, a further key recommendation is the need to use clear and fitting communication. While communication that is tailored to the needs of the individual is preferred, there is also a role for communication that is targeted and embedded within the community, including posters and TV adverts. These mediums should incorporate trusted sources and messages would need to be designed together with the target group to ensure they are appropriate.

A further consideration is the literacy levels of the target population. It has been found that low literacy levels cause difficulty for 1 in 4 adults in Scotland [51]. Our results indicated people may be less inclined to complete a survey than to get a blood test done, with low literacy being one reason for this. Similarly, to mitigate mentioned difficulties reading test invitations and information, the use of a phoneline was suggested. Thus, any future lung cancer screening programme should be mindful of the expected literacy capabilities of the target populations and employ communication strategies that reduces this barrier, which will enable increased uptake in this population.

One final consideration regarding language is the way the programme is described or named. Previous research has trialled the term “M.O.T for your lungs” and considered “Lung Health Check”, however, these are expected to have varying degrees of impact [23, 52]. Using such terms was suggested as a possible means to overcome guilt associated with smoking in our study and ranked reasonably high as a possible facilitator, indicating there is possibly further research needed on this concept.

#### Overcoming barriers with options

In summary, it was clear that a one-size-fits-all approach is likely to be unsuccessful. Individuals in the target population may have competing priorities and therefore it is key to empower and enable participants to participate, whether that is in a smoking cessation clinic, routine care, local drop in, GP appointment, at work or in a mobile unit. Providing a range of options could lead to increased uptake. Also, this work identified some priorities, more work is required to determine which combination of options would be cost-effective.

### Strengths & limitations

Participants recruited represented both target populations (Professionals and high-risk population). Although few participants met all three high-risk indicators for lung cancer (aged 55 + , ever-smoker, living in area of deprivation), all met at least one and all indicators were represented in the sample. In Phase 1 the majority of participants were male, but this was balanced out by a greater number of female participants in Phase 2. Findings should be generalised with care as ethnicity data were not collected. Due to the timing in the COVID-19 pandemic, fewer professionals were able to participate in Phase 2 than anticipated. However, the views of the respiratory consultants who were consulted in Phase 1 were carried forward to Phase 2, in line with the co-design process. Collaboration had to take place remotely due to social distancing, which partly took place online and partly offline by liaising with community leads to ensure that all stakeholders could comfortably engage.

### Next steps

Continuing to work together with relevant stakeholders to develop the solutions set out in this article will be key to the success of a future lung cancer screening programme. For programmes making use of biomarker blood tests, we specifically suggest the co-design of a national and targeted advertising campaign, communication and information regarding test use and programme participation, training packages for professionals and the development of an associated biorepository to ensure acceptability and usability. Further work is also needed to improve the usability of the home test kit, whether through redesign, accessible instructions or support provision. High-risk groups in areas of high deprivation should continue to be involved in future development steps, using accessible and appropriate engagement methods, to ensure that resulting solutions meet their needs.

## Conclusion

In all, this study has provided a fuller understanding of the barriers, facilitators and pathways for the provision of a future lung cancer screening programme, especially regarding the use of biomarker blood test for the identification of at-risk individuals. Ten barriers: Fear of result and impact (B1), Guilt & stigma around smoking (B2), Attitude/apathy (B3), Mental health and anxiety (B4), Hesitation around Covid-19 (B5), Lack of engagement with healthcare (B6), Lifestyle (B7), Travel (mobility and rurality: B8), Literacy (B9), Number of interactions (B10) and 8 facilitators: Perceived benefits (F1), Trust in HCPs (F2) Immediacy (F3), Part of the community (F4), Convenience and options (F5), Embedded conversations in the community (F6), National advertising campaigns (F7), Trusted sources (F8) to uptake were identified. A further four barriers: Staff capacity (PB1), Attitude about the blood test and uptake (PB3), Complications due to COVID 19 (PB2), Hesitations around logistics and follow up (PB4) and four facilitators: Buy-in (PF1), Financial incentives (PF2), Optimally streamlined, ensuring capacity (PF3), Training (PF4)-to provision were found.

Moreover, the insights of this study led to three key recommendations to improve uptake to a future lung cancer screening programme amongst those who could benefit most: 1. Embedding the service in communities, 2. Effective communication and 3. Overcoming barriers with options. This along with the findings regarding the acceptability of an associated biorepository should aid the development of an acceptable and effective future lung cancer screening programme, meeting the needs of those who could benefit most. Using co-design to further develop this programme will help to ensure that lung cancer screening will reach those who could benefit most.

## Data Availability

The datasets used and/or analysed during the current study are available from the corresponding author on reasonable request.
